# BDNF as a Putative Target for Standardized Extract of *Ginkgo biloba*-Induced Persistence of Object Recognition Memory

**DOI:** 10.3390/molecules26113326

**Published:** 2021-06-01

**Authors:** Beatriz G. Muratori, Cláudia R. Zamberlam, Thaís B. Mendes, Bruno H. N. Nozima, Janete M. Cerutti, Suzete M. Cerutti

**Affiliations:** 1 Cellular and Behavioral Pharmacology Laboratory, The Graduate Program in Biological Chemistry, Universidade Federal de São Paulo, São Paulo 09972-270, Brazil; muratoribeatriz@gmail.com (B.G.M.); crzamberlam@gmail.com (C.R.Z.); 2 Genetic Bases of Thyroid Tumor Laboratory, Division of Genetics, Department of Morphology and Genetics, Federal University of Sao Paulo, São Paulo 04039-032, Brazil; thais_biude@hotmail.com (T.B.M.); bruno.nozima@gmail.com (B.H.N.N.); j.cerutti@unifesp.br (J.M.C.); 3 Department of Biological Science, Universidade Federal de São Paulo, São Paulo 09972-270, Brazil

**Keywords:** memory, object recognition, *Ginkgo biloba*, dorsal hippocampus formation, brain-derived neurotrophic factor

## Abstract

Despite considerable progress on the study of the effect of standardized extract of *Gingko biloba* (EGb) on memory processes, our understanding of its role in the persistence of long-term memory (LTM) and the molecular mechanism underlying its effect, particularly episodic-like memory, is limited. We here investigated the effects of EGb on the long-term retention of recognition memory and its persistence and BDNF expression levels in the dorsal hippocampal formation (DHF). Adult male Wistar rats (n = 10/group) were handled for 10 min/5 day. On day 6, the animals were treated with vehicle or 0.4 mg/kg diazepam (control groups) or with EGb (250, 500 or 100 mg/kg) 30 min before the training session (TR1), in which the animals were exposed to two sample objects. On day 7, all rats underwent a second training session (TR2) as described in the TR1 but without drug treatment. Object recognition memory (ORM) was evaluated on day 8 (retention test, T1) and day 9 (persistence test, T2). At the end of T1or T2, animals were decapitated, and DHF samples were frozen at −80 °C for analyses of the differential expression of BDNF by Western blotting. EGb-treated groups spent more time exploring the novel object in T2 and showed the highest recognition index (RI) values during the T1 and T2, which was associated with upregulation of BDNF expression in the DHF in a dose-and session-dependent manner. Our data reveal, for the first time, that EGb treatment before acquisition of ORM promotes persistence of LTM by BDNF differential expression.

## 1. Introduction

Flavonoids (isoflavones, flavanones, anthocyanins, flavanols, flavones and flavonols) are bioactive molecules from plants or foods that, along with non-flavonoid compounds (phenolic acids and other phenols), are widely used to prevent or treat cognitive deficits. Extracts rich in flavonoids or flavonoid fractions have been associated with improvements in memory formation and stress-related memory processes [1,2,3]. A recent study showed that standardized extract of *Ginkgo biloba* (EGb), which is obtained from the dried leaves of the plant and is standardized to contain 24% to 27% ginkgo-flavonoglycosides and 6% ginkgo-terpenoid lactones [4], increases BDNF expression, which has a protective effect on neurons [5,6,7]. Standardized extract of *Ginkgo biloba* (EGb) has been used as a therapeutic alternative for the treatment of Alzheimer’s disease and multi-infarct dementias and has significant positive effects on cognitive function in patients with Alzheimer’s disease [8,9]. Similar results have been observed after Ginkgo terpene trilactone (GTTL) treatment. GTTLs increase BDNF expression in the hippocampus, which protects hippocampal neurons against epilepsy and improves spatial memory, as evaluated by the Morris Water Maze (MWM) test [5,10].

Studies from our group have shown that acute or chronic treatment with EGb modulates fear memory formation, which is associated with increased levels of cAMP-dependent response element binding protein (CREB) in the hippocampal formation [11,12]. Conversely, our group reported an impairment in conditioned fear memory following diazepam treatment, which was associated with reduced CREB-1 expression (both mRNA and protein) in the dorsal hippocampus [11]. In addition, we showed that the mRNA and protein expression of the serotonin type-1A (5-HT_1A_) receptor and the GluN2B and GluN2A subunits of the N-methyl-D-aspartate (NMDA)-type glutamate receptor in the dHF are modulated after EGb treatment and fear memory acquisition [13]. We also showed that flavones modulate fear memory formation and persistence through gamma-aminobutyric acid type A receptor (GABA_A_R) modulation [14,15]. Furthermore, GABA_A_R agonists impair the expression of object recognition memory (ORM), which seems to be correlated with BDNF expression and the persistence of LTM [16].

The ability of an animal to recognize a familiar object using the physical characteristics of the stimulus or the contextual information concerning the object (spatial and/or temporal order) involves discriminative memory formation. Recognition memory for an object or object-in-context in animals has been termed episodic-like memory and reflects an experience-dependent internal representation of what happened and where and when the event occurred. When the original memory is re-experienced, we recall details of the experience [17,18,19,20]. In this regard, recognition memory formation involves both familiarity and recollection of an episode, i.e., quantitative or qualitative information about a previously encountered item or event [21]. Both the familiarity and recollection components become less persistent over time [22,23].

The ORM task has been employed to investigate the effects of drug treatments on memory as well as the neuroanatomical, neurophysiological and neurochemical changes underlying spontaneous exploratory behavior in animals [24,25,26]. Numerous studies have explored the contribution of the perirhinal and parahippocampal cortices and the hippocampal formation (HF) to familiarity and recollection [18,27,28,29,30,31]. Recent advances have led to substantial improvements in our understanding of the role of the hippocampus in processing and integrating multisensory information and encoding long-term novel object recognition (NOR) memory, novel location memory (NLM) or temporal order memory (recency) [17,18,29,32,33,34], but the debate undoubtedly continues [35]. Moreover, the role of the hippocampus in the reconsolidation and persistence of ORM as well as molecular changes underlying these processes has been shown [16,36,37,38].

It was recently shown that reduced expression of brain-derived neurotrophic factor (BDNF) in the hippocampus is associated with impaired long-term ORM [39]. Activity-dependent secretion of BDNF has been associated with both hippocampus-dependent long-term memory formation and long-term biochemical and morphological dendritic changes [40,41] since it is crucial for long-term memory consolidation [42,43]. A growing body of evidence has shown that BDNF overexpression and activity modulate the differentiation, maturation, survival and maintenance of different populations of neurons and glial cells and has identified the main effects of BDNF on short- and long-term memory-related processes [44,45] and neurogenesis [46]. *De novo* synthesis of BDNF has been shown to be essential and sufficient for promoting late-phase long-term potentiation (L-LTP) [47] and improving long-term memory (LTM) [48]. Increased BDNF expression (mRNA and protein) in the hippocampus (1 or 12 h after training, respectively) is critical for LTM formation in a hippocampus-dependent acquisition task [24]. In addition, BDNF is a key molecule for the prevention of memory loss, the generation of persistent fear and long-lasting memory traces and the extended maintenance of LTM [22] and is also required for reconsolidation of NOR memory [16,49].

Together, these data show that BDNF/TrKB receptor-induced synaptic plasticity, survival and dendritic and axonal growth during hippocampal-dependent memory processes are mediated by distinct signalling pathways in both pre- and post-synaptic cells, which involve increases in extracellular signal-regulated protein kinase (ERK) expression and phosphorylation and activation of cyclic AMP response element-binding protein (CREB) [22,50,51].Despite the findings of these studies, little is known about novel therapeutic strategies for modulating the cellular and molecular processes that mediate the persistence of recognition memory. Flavonoids have long been targeted as prospective therapeutic agents for memory improvement [1].

Considering recent findings from our group describing the effects of EGb on the persistence of fear memory [52,53], we investigated the effects of the administration of EGb before the acquisition of object recognition memory (ORM), a non-aversive memory task, on the long-term retention of ORM and its persistence. Episodic-like memory loss is correlated with both early-stage Alzheimer’s disease (AD) and normal aging. We wondered whether rats treated with EGb would have improved their original memory, i.e., does EGb affect memory consolidation and promote object recognition memory persistence? We analysed retrieval and within-session maintenance of ORM during the T1 phase, as well as the persistence of memory in the T2 phase. We also examine potential roles for diazepam, a GABA_A_R agonist, in modulating the acquisition of non-aversive memory and in mediating memory retention and persistence through alterations in BDNF expression. Last, given the pivotal role of BDNF in the neurochemical and morphological changes associated with LTM and the persistence of LTM, we analysed BDNF expression in the dorsal hippocampal formation of the control and EGb-treated groups after retention and persistence tests.

## 2. Results

### 2.1. Behavioral Analysis

**Figure 1:**
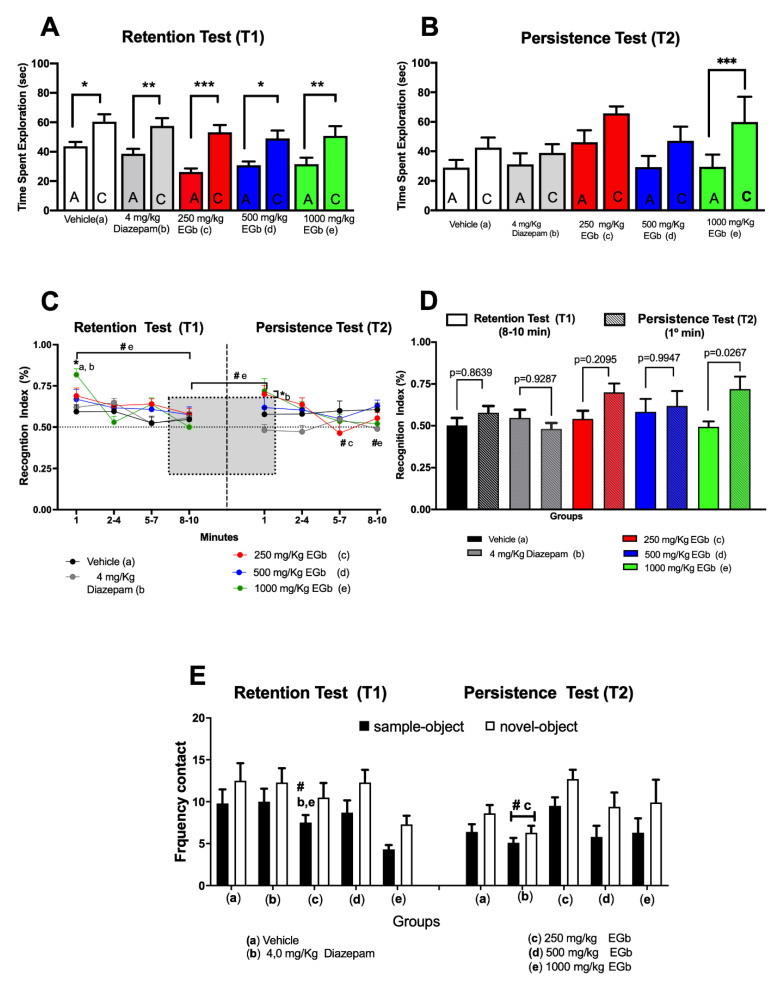
(**A**). Comparison of the mean of total time spent exploring/min the novel and sample objects in the retention test (day 8, n = 20/group), and the persistence test (**B**) (day 9, n = 10/group) between the control (diazepam- and vehicle-treated) and EGb-treated (250, 500 and 1000 mg/kg) groups. (**C**). The recognition index (RI) during the retention and the persistence sessions. The first points indicate the mean RI values during first min of the control (4 mg/kg diazepam- and vehicle-treated) and EGb-treated (250 mg/kg, 500 mg/kg and 1000 mg/kg) groups before the acquisition of ORM (n = 20/group) and the persistence test session (n = 10/group). The subsequent data points represent the mean DI values of nine min in blocks of 3 min (trials) each (2–4,5–7,8–10). EGb, diazepam and vehicle were administered orally 30 min prior to training session. No drugs were administered before retention test (T1) and persistence test (T2) sessions (**D**). Persistence of long-term memory (LTM) was inferred based on a comparison of the time spent spontaneously exploring sample object and that spent spontaneously exploring the novel object during the last three trial blocks at the end of the retention session and during the first trial of the initial persistence session. (**E**). Frequency of contact with the sample and novel objects during the retention test (n = 20/group) and persistence test (n = 10/group) for the groups treated with EGb (250 mg/kg, 500 mg/kg or 1000 mg/kg), diazepam or vehicle (n = 10/group). Analyses of the sample vs novel object and group interaction, the time and group interaction (A, B, C, and D panels, respectively) and one random factor (rat) was performed using GraphPad Prism Software^®^. The values are presented as the means/min (± SEMs) (A, B, D, and E) and/or a block of three minutes (±SEMs) (**C**). * *p* < 0.05, ** *p* < 0.01 and *** *p* < 0.001; two-way repeated measures ANOVA with the *post hoc* Sidak multiple comparison test (**A**,**B**). Two-way repeated measures ANOVA was conducted for within- and between-group comparisons followed by Tukey’s multiple comparisons test (**C**). Within-group comparison # *p* < 0.05; two-way ANOVA followed by Bonferroni’s *post hoc* test (**D**,**E**). Comparisons between groups are presented * *p* < 0.05, ** *p* < 0.01 and *** *p* < 0.001.

To examine the cognition-enhancing effect of EGb on long-term memory for novel objects in rats, we employed two 10-min sessions (T1 and T2) of the NOR test, a non-aversive task, with an intertrial interval of 24 h. Memory formation and memory persistence were assessed by different analyses of total spontaneous exploration time for both objects in the T1 and T2 sessions (Figure 1).

Two-way RM ANOVA, indicated significant main effects of time exploring the familiar and novel objects (F_1__.95_ = 59.84; *p* < 0.0001) and effects of group (F_4__.95 _= 2.967; *p* = 0.0234) but no interaction between object and group (F_4__.95_ = 0.4771; *p* = 0.7524). Comparative analyses of the mean total time spent (min) exploring each of the objects during the retention session (T1) between and within groups showed that the control groups (vehicle- and diazepam-treated group; n = 20 rats/group) and EGb-treated groups (250, 500 and 1000 mg/kg EGb-treated groups; n = 20 rats/group) spent more time exploring the novel object than the familiar object in the T1 session (Figure 1A). Based on our data, all rats recalled the familiar object and preferred to explore the novel object.

During T2 session when the animals were re-exposed to the objects, Two-way RM ANOVA indicated significant effects of time spent exploring the familiar object vs novel objects within groups (F_1__.45_ = 31.12; *p* < 0.0001). Bonferroni’s multiple comparisons test revealed the rats that had been treated with 1000 mg/kg EGb spent more time exploring the novel object than the familiar object compared with the vehicle- and diazepam-treated groups, as shown in Figure 1B. However, no interaction was observed between the factors group and object (F_1.45_ = 1.385; *p* = 0.2549) and no difference was observed between groups (F_4.45_ = 1.144; *p* = 0.3496).

Additionally, the time spent interacting with the sample object (A) and the novel object (C) were used to calculate the recognition index [RI = time spent exploring the novel object/total object exploration time (novel vs familiar)] [28,54,55]. RI represents a relative measure of discrimination between objects. The EI values in each session (T1 and T2) are shown in Figure 1C. The RI value in the first minute is shown for each session since the effects of novelty might decrease over time. The subsequent data *points* are the *mean RI value over a* three-minute period within a given session (three blocks of 3 min each as follows: trial 2–4 min; trial 5–7 min; and trial 8–10 min). Furthermore, within-session comparisons (min-by-min) at T1 using two-way RM ANOVA showed an interaction between groups and time (F_36, 855_ = 1.756; *p* = 0.0043, with a difference only in the first minute in relation to subsequent times (min 2–10) (F_7.9833, 758.4_ = 1.914; *p* = 0.05), as well as effects of the groups (F_4, 95_ = 43.572; *p* = 0.0093). Concerning the min-by-min analysis of the recognition index (RI) in T2, two-way RM ANOVA showed no interaction between groups and time (F_36, 405_ = 1.411; *p* = 0.06), no within-session difference (min-by-min), i.e., first minutes in relation to subsequent minutes (2–10) (F_7.706, 346.8_ = 1.581; *p* = 0.1320) and no effects of the groups (F_4, 45_ = 0.5821; *p* = 0.6772). Thus, this procedure evaluates the mean of a combination of 3 min to help reduce variance within sessions and has been systematically used in our lab since it allows us to evaluate retention of short- and long-term memory. An RI value of 1.0 indicates that the animal spent the majority of time exploring the novel object relative to the sample object, and a value of ≤0.5 indicates random exploration, i.e., no discrimination between objects or decreasing novelty. Two-way repeated-measures ANOVA followed by Tukey’s multiple-comparison test revealed that all groups spent more time exploring the novel object than the previously explored (familiar) object (250 mg/kg EGb, RI_1_ = 0.66; 500 mg/kg EGb, RI_1_ = 0.66; 1000 mg/kg EGb, RI_1_ = 0.81; vehicle, RI_1_ = 0.59; 4 mg/kg diazepam, RI_1_ = 0.61) There was an interaction between group and time (F_12.285_ = 3.045; *p* = 0.0005) and a main effect of time (F_3.285_ = 11.76; *p* < 0.0001) for the T1 session (Figure 1C, left panel). Moreover, the high-dose EGb-treated group spent more time exploring the novel object than the control groups (diazepam- and vehicle-treated groups). Furthermore, an intragroup comparison of the 1000 mg/kg EGb group showed that the RI in the first minute was significantly lower than that in any of the all three-minute blocks. Although our data showed difference between in first minute and last three minutes during T1, the RI was maintenance > 0.5, indicating that within-session the animals treated with 1000 mg/kg EGb spent more time exploring novel object. Because vehicle and EGb treatments were given only before TR1, our data suggest that 1000 mg/kg EGb improved the original memory, i.e., memory for early familiar object (object A) in relation to late object (object C). Our present data is consistent with previous results from our lab that show effects of EGb on persistence of fear memory [52,53].

Memory persistence was analyzed based on the data obtained in the T2 session (Figure 1C, right panel). Two-way RM ANOVA revealed no interaction between group or trial (F_12.135_ = 1.49; *p* = 0.133), but there was a main effect of group (F_4.45_ = 2.932; *p* < 0.0308). Rats treated with EGb (250 mg/kg, RI_1_ = 0.69; 500 mg/kg, RI_1_ = 0.61; 1000 mg/kg, RI_1_ = 0.71) and vehicle (RI_1_ = 0.57) spend more time exploring the novel object than the familiar object during the first minute. Conversely, rats treated with diazepam spend similar amounts of time exploring both objects (RI_1_ = 0.48). Tukey’s multiple comparison test revealed a significant decrease in the EI in the diazepam group compared to the 250 mg/kg EGb (*p* < 0.05) and 1000 mg/kg EGb (*p* < 0.01) groups. Analysis of the within-session data showed that the RI in the first minute was significantly lower than that in the second three-minute block for the 250 mg/kg EGb-treated group at (*p* < 0.01) and the third three-minute block for the 1000 mg/kg EGb group. The mean (and S.E.M) of the RI of the EGb-treated and control groups in both sessions are presented in the supplementary data (Table S1).

Moreover, to substantiate our results regarding the effects of EGb on the persistence of LTM, we also investigated recognition index (RI) between sessions by comparing the mean RIs from the first minute of the persistence test session with those from the last block of the three min of the retention test (Figure 1D). Two-way ANOVA revealed no interaction between group and session (F_4.45_ = 2.095; *p* = 0.0969), no effect of group (F_4.45_ = 1.187; *p* = 0.3292), and a main effect of time (F_1.45_ = 6.249; *p* = 0.0161).

Our present data corroborate previous findings and shows that rats treated with 1000 mg/kg EGb exhibit a higher RI during the first trial of the T2 session than during last three-minute block in the T1 session, indicating maintenance of the original memory, i.e., the information regarding the object appeared to be preserved (old vs. novel) 48 h after acquisition (T2).

Because a number of studies have shown that animals have preferences for any one object [56,57], we also examined the frequency of contact with each sample and novel object (Figure 1E). Two-way RM ANOVA revealed no interaction between the group and object in the T1 session (F_4.72_ = 0.05093; *p* = 0.9950) but showed main effects of frequency of contacting the object (F_1.18_ = 9.996; *p* = 0.0055) and treatment (F_3.237, 58.27_ = 4.573; *p* = 0.0050). 

Our data showed that all groups contacted both objects with a similar frequency. Tukey’s multiple comparisons test indicated a significant difference in the frequency of contact with the sample object between the 1000 mg/kg EGb group and the diazepam group. Comparative analyses of the frequency of contact with both the familiar and novel objects in the T2 session revealed no interaction between the group and object (F_4.36_ = 1.082; *p* = 0.3800) and no effect of treatment (F_4.36_ = 2.454; *p* = 0.0633), but a main effect of frequency of contact with both objects (F_1.9_ = 36.46; *p* = 0.0002). The EGb-treated groups exhibited a higher frequency of contact with the novel object than the familiar object. No differences were observed in the diazepam and vehicle groups.

**Figure 2:**
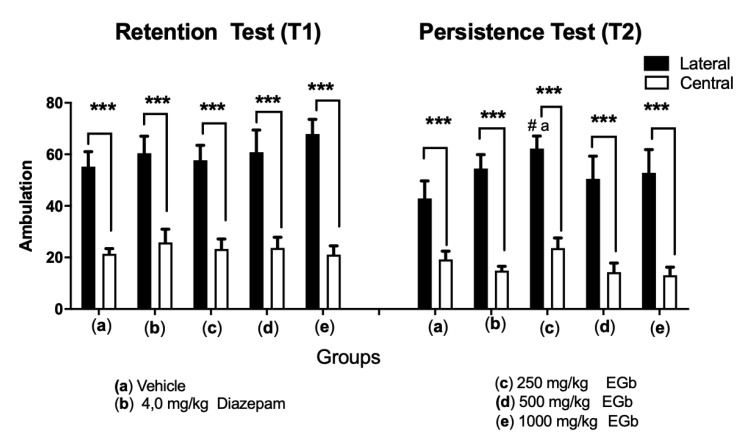
Number total of central and lateral square crossings (ambulation) in the control groups (4 mg/kg diazepam- and vehicle-treated group) and the groups treated with EGb (250 mg/kg; 500 mg/kg and 1000 mg/kg) during the retention test (n = 20/group) and persistence test (n = 10/group). The values are expressed as the mean values/ min (± SEMs). *** *p* < 0.0001; two-way ANOVA followed by post hoc Tukey’s multiple comparison test. # *p* < 0.05 denotes a difference within groups. between sessions comparisons.

Additionally, we investigated exploratory behaviors such as the amount of ambulation (lateral and central squares) and rearing and grooming of all animals during the retention test and persistence test (Figure 2). The data showed significantly increased ambulation in the lateral squares compared with the central squares in both the retention session (F_1.18_ = 266; *p* < 0.0001) and persistence session (F_1.18_ = 6.663; *p* = 0.0191). Two-way RM ANOVA revealed no interaction between group and area (F_4.72_ = 0.4444; *p* = 0.7761) and no effect of treatment (F_2963, 53.33_ = 0.3472; *p* = 0.7875) in the T1 session. Similarly, no interaction between group and area (F_4.72_ = 0.6991; *p* = 0.5951) and no effects of treatment (F_2.941, 5293_ = 1.352; *p* = 0.2676) were found in the T2 session (Figure 2).

One-way RM ANOVA indicated that the total level of rearing behavior was similar for all groups in the T1 (F_4.45_ = 2.424; *p* = 0.0619) and T2 session (F_4.45_ = 0.8002; *p* = 0.5315). All data are shown as the means ± SEMs in Supplementary Table S2.

Finally, we examined the level of grooming, and the two-way RM ANOVA showed an effect of time (F_4.45_ = 1647; *p* = 0.1790) in T1 and in T2 session (F_4.45_ = 0.7617; *p* = 0.5557). All data are shown as the means ± SEMs in Supplementary Table S2.

### 2.2. Molecular Analysis

We also evaluated the levels of the BDNF protein in relation to tubulin in the hippocampal formation after the retention and persistence sessions. Compared with no treatment (naïve group), treatment with all doses of EGb before the acquisition of object recognition memory upregulated BDNF expression in animals subjected to ORM retrieval in the retention test. One-way ANOVA revealed that a main effect of the treatment (F_5.21_ = 5.810; *p* = 0.0016) on performance in the retention test.

**Figure 3:**
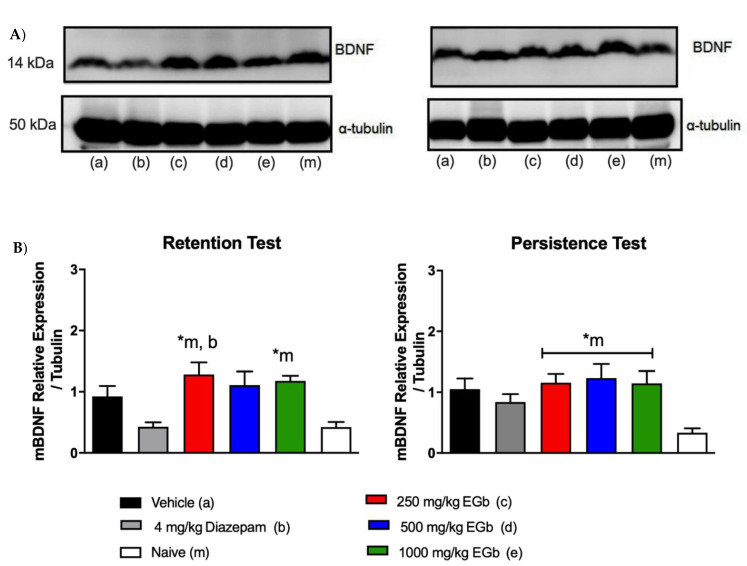
(**A**). Representative Western blot images. (**B**). Quantitative analysis of BDNF and α-tubulin expression in homogenates of the hippocampal formation obtained from the control (the 4 mg/kg diazepam (b), vehicle (a) and naïve (m) groups and the groups treated with EGb (250 mg/kg (c), 500 mg/kg (d) or 1000 mg/kg (e)), as determined by immunoblotting. The data were normalized to the level of α-tubulin in the retention and persistence test sessions (n = 5 rats/group). The values are presented as the mean values (± SEMs). * *p* < 0.05 one-way ANOVA followed by *post hoc* Tukey’s multiple comparison test.

Furthermore, increased levels of BDNF were observed in the 250 and 1000 mg/kg EGb-treated groups compared with the naïve group in the T2 session (F_5, 23_ = 4.005; *p* = 0.0093) (Figure 3). Our data in line with previous data about key role of BDNF on persistence of memory [18], since control and 500 mg/kg EGb groups didn’t have differential expression of BDNF during retrieval of memory, which suggest that upregulation of BDNF during both sessions is required to persistence of memory.

## 3. Discussion

Our present data show that all groups spent more time exploring the novel object than the familiar object in the retrieval (T1) session, but only the EGb-treated group at dose 1000 mg/kg spent more time exploring the novel object during the persistence session (T2). In addition, discrimination ability was evaluated within and between sessions by employing the recognition index (RI). Within-session analysis showed that RI was higher in the 1000 mg/kg EGb-treated group than the vehicle and diazepam groups during the first minute of the retrieval test. Similarly, the modulatory effect of EGb was observed during re-exposure of the animals to the same objects. Rats treated with 250 or 1000 mg/kg EGb had the highest RI values during the persistence session. The maintenance of memory for “novel” and familiar objects 96 h after acquisition of ORM was observed in the EGb-treated groups but not in the other groups.

Our data also revealed that rats treated with diazepam exhibit poor performance during the T2 session because the animals spent similar amounts of time exploring both the novel and familiar object, exhibiting an RI < 0.5. A reduced ability to recollect a previously experienced item suggests that diazepam prevented the persistence of ORM. To assess the effects of EGb on the persistence of ORM, we compared the RI data from the first min with those from the last three min of the retention test.

These data substantiate the findings regarding the total exploration time since the rats treated with the high dose of EGb exhibited an DI value similar to that observed during the first trial of the retention test. These effects suggest that EGb treatment led the animals to favor the innate novelty of the object, as reported in the literature [58]. Moreover, the number of line crosses in the central and lateral squares in the open field arena (Figure 2) and the levels of rearing and grooming (Supplementary Table S2) within sessions were evaluated to investigate whether or not the rats exhibited changes in spontaneous exploratory behavior or some aspects of emotionality including anxiety, which might lead to changes in object exploration and thus impair recognition memory. Based on the number of squares crossed, all groups of treated rats preferred to enter the lateral squares rather than the central squares, as the lateral squares elicited less anxiety than the central squares. Thus, EGb did not have an effect against anxiety. However, the size of open-field arena was chosen to reduce the levels of anxiety/aversion and to prevent potential interference with object exploration [59].

Because no difference in these behaviors was found between the treated groups and the control group (vehicle), it is unlikely that the findings observed following EGb or diazepam treatment were associated with motivation to explore or motor impairments. In addition, we evaluated the number of times (the frequency) the animals contacted each object since the number of contacts is a potential indicator of object recognition memory. During the T1 session, all groups had exhibited an increased frequency of contact with the novel object compared to the familiar object. However, during the T2 session, only the EGb-treated groups spent more time contacting the more recently encountered object than the familiar object.

Taken together, these results corroborate previous data from our group showing that that treatment with EGb improves long-term memory in a dose-dependent manner, as assessed by conditioned suppression of licking behavior as well as modulation of the spontaneous recovery of conditioned fear [11,12,13,52]. Here, we reveal a relationship between acute EGb treatment and the persistence of non-aversive memory for the first time. EGb-treated rats exhibit improvements in the recovery of memory for objects (what) and temporal order of the items (when). Moreover, we showed that maintenance of ORM caused by EGb treatment was associated with upregulation of BDNF in the dorsal hippocampal formation. However, no difference was detected in BDNF protein expression in the dHF between the vehicle, diazepam and naïve groups.

As mentioned in the introduction, novelty detection is associated with the hippocampus [60] and entorhinal cortex [61]. Here, we found that EGb treatment resulted in upregulation of BDNF expression in the dorsal hippocampal formation 72 and 96 hr after acquisition of ORM, which was associated with the persistence of ORM and was not seen in the control group [49,62,63].

Studies have shown that low levels of BDNF impair memory formation in animals subjected to an object recognition task. Reduced expression of BDNF in the hippocampus and parietal, entorhinal and frontal cortices results in noticeably reduced plasticity and cognitive impairment, which are correlated with progression of neurodegeneration and dementia [64]. In addition, it has been shown that EGb treatment can significantly enhance the levels of BDNF expression in the mouse hippocampus, leading to reversal of neural damage induced by acrylamide treatment [39]. The critical role of BDNF in the persistence of fear memory was previously described. Increased expression of BDNF may reverse memory deficits [22].

At the molecular level, BDNF is a modulator with a well-known effect on synaptic transmission and plasticity in different areas of the central nervous system [65], which contribute to the adaptative processes involved in consolidation of memory. CREB expression is modulated by multiple signalling pathways, including pathways associated with PKA-MAPKs, CaMK, and N-methyl-D-aspartate receptors (NMDA-Rs), which can converge to increase BDNF expression and modulate cytoskeleton protein synthesis and anti-apoptotic activity and are involved in molecular adaptation processes such as dendritic growth and ramification and the formation of stable long-term potentiated synapses necessary for consolidation processes.

Interactions of these different pathways, which converge on CREB and BDNF expression, are thought to be crucial for the effects of flavonoids on memory enhancement [1,66]. In previous studies from our group, we analysed the neural circuits and neurochemical changes following EGb treatment and fear memory formation [13,15,57,60] as well as the effects of crude extract of erythrina (CE), flavonoidic fraction or isolated compounds from CE on conditioned suppression [14,67]. EGb treatment prior to conditioning was subsequently evaluated after 72 or 96 h during the extinction retention test, and EGb was demonstrated to have effects on the spontaneous recovery of extinction. Furthermore, we showed that EGb treatment (acute or chronic) before conditioning increased the levels of CREB expression in the dorsal hippocampal formation. These data suggest that CREB expression might be correlated with the upregulation of BDNF observed in the present study. Although further studies are needed to better identify the mechanisms underlying object recognition memory, our data show for first time that BDNF in the dorsal hippocampal formation is an important target of EGb in promoting the persistence of object recognition memory.

## 4. Materials and Methods

### 4.1. Animals

A total of 120 experimentally naïve male Wistar rats (10 to 12 weeks old) were obtained from the Center for the Development of Experimental Medicine and Biology (Universidade Federal, São Paulo, Brazil). The animals were housed four per cage and provided food and water ad libitum during the acclimatization period (15 days) and experimental procedure. The animals were kept at a controlled temperature (21 °C ± 3) and relative humidity (55 ± 10) on a 12-h light/dark cycle (lights on at 6:00). The experiments were performed during the light cycle. All experimental procedures were approved by the local Committee Governing the Ethics on the use of Animal Experimentation of the Federal University of Sao Paulo (CEUA Unifesp 3447100417) and were conducted in accordance with the national animal care legislation and guidelines (Brazilian Law 11794/2008), as suggested by the APA Guidelines for Ethical Conduct in the Care. The animals were randomly assigned to control and EGb-treated groups. The animals in the control groups were distributed into the following three subgroups (n = 20/group): (I) the naïve group (no training/no treatment; protein expression control); (II) the vehicle group (0.9% saline; control) and, (III) diazepam-treated group (4 mg/kg, negative control). The animals in the EGb groups were further divided into subgroups according to the dose administered: (I) 250 mg/kg; (II) 500 mg/kg, or (III) 1000 mg/kg (n = 20/group).

### 4.2. Drug Administration

A previously described standardized *Ginkgo biloba* extract obtained from the green leaves of *Ginkgo biloba* (EGb) that contained 24% ginkgo-flavoglycosides and 6% ginkgo-terpenoid lactones [52] was used in all experiments. This extract has a formulation and composition identical to the products registered in Germany (Dr. Willmar Schwabe Pharmaceuticals, Karlsruhe, Germany). The dose range of EGb was chosen according to previous studies conducted in our laboratory [11,12,15,60]. According to previous data from our group, 4 mg/kg diazepam impairs the acquisition of fear memory in rats, and for this reason it was used as a negative control. EGb and diazepam were re-suspended in saline 0.9% (vehicle) and administered orally via an intragastric (IG) tube 30 min before the training session (TR1). No substances were administered in the other trial sessions. 

### 4.3. Apparatus and Objects

The novel object recognition (NOR) task was conducted in a white square arena made of wood (40 × 40 × 40 cm), the floor of which was divided into different sections (central and lateral squares). A video camera fixed 100 cm above the apparatus was used to record all sessions for behavioral analysis. The objects used as the “sample objects” (old objects) for the training session and as the “novel object” (recent object) for the test session were chosen according to weight and size based on the literature [58]. The sample objects were always placed in the same corner (right or left) of the open field and positioned at the same place in the room to allow the use of distal cues. To minimize bias resulting from individual preference for the specific objects, we chose objects with a similar texture and colour pattern. In both the familiarization and test phases, rats were individually placed at the midpoint of the opposite wall of the box facing the wall to prevent coercing the rats to explore the objects. All experimental sessions were conducted using one rat from each group (control or treated). After each rat was tested, the apparatus and objects were carefully cleaned with 10% ethanol solution to remove odour cues [3,54,55].

### 4.4. Novel Object Recognition (NOR) Procedure

The testing protocol consisted of three phases (4 consecutive days): habituation, familiarization (two days) and the test phase (two days). All behavioral sessions were carried out between 8 a.m. and 1 p.m., including habituation of the animals to the testing room and to the researchers (the handling period).

#### 4.4.1. Handling

Prior to beginning the experimental sessions, all animals were subjected to handling for a period of 20 min/day over 5 days (~2 min per rat each day) to avoid the effects of excessive handling after transport, as the novelty of handling has been shown to affect the acquisition of memory [58]. For the NOR test, the rats were individually transferred to and maintained in the adjacent room under light of a controlled intensity for 10 min before the sessions (Figure 1A).

#### 4.4.2. Acquisition

On the 6th and 7th day all rats except those in the naïve group were allowed to freely explore the sample objects in order for 15 min to allow habituation to the objects (sample objects A and B) and the arena. This was considered the familiarization phase (F1 and F2) and is when the acquisition of object recognition memory occurs [68]. To prevent bias related to object exploration, the rats were placed against the centre of the opposite wall with their back to the objects. After the animals were placed in the open field arena, the experimenter left the room to avoid interfering with the animal’s behavior (Figure 1B,C). Rationale for choosing of the familiarization procedure used in this study was based on previous data from the literature, such as the study by Shimoda and colleagues. [69], which showed no difference between the length of familiarization in the sample phase (5, 15 and 30 min) and subsequent novelty recognition performance at the test phase with a 24-h delay period. Complementarily, data from the study by Ozawa and colleagues suggest that longer exploration of the objects in the familiarization period may allow animals to make associations between each element of information, such as objects [70]. Moreover, another study revealed that a sample session of 10 min each for three days supports the encoding of strong object memory [60,71], and promotes persistence of object memory. Consistent with these data and previous data from our lab showing the effects of EGb as a cognitive enhancer [13,14,56,57] we hypothesized that associated effects, such as a longer sample phase and EGb treatment, would improve recognition memory formation and support the persistence of long-term recognition memory in rats in the object recognition test (ORT), since both the familiarity and recollection components become less persistent over time [22,23].

In addition, we counterbalanced the objects serving as A and B (e.g., a bottle served as object A and a statuette served as object B for half of the rats; the pattern was reversed for the remaining rats). Furthermore, the two objects had similar patterns (e.g., size and no odour cues). As defined by Ennaceur and Delacour [72], the exploration of an object was considered when the rat directed its nose at a distance ≥ 2 cm to the object and/or touched it with the nose, while turning around or sitting on the object was not considered an exploration.

#### 4.4.3. Retention Test

All rats except those in the naïve group, were returned to the same chamber for analysis of long-term memory (LTM) for the objects (8th day) (n = 20/group) (Figure 1A–E). Each rat underwent a test trial that was identical to the second familiarization session except that one object was identical to the sample trial (object A or B) and one object was the novel object (object C). Twenty-four h after the end of the T1 session, half of the rats were euthanized (n = 10/group). The remaining half (n = 10/group) was subjected to the persistence test to evaluate the effects of EGb on the maintenance of memory.

#### 4.4.4. Persistence Test

Previous data from the literature showed that long intervals of time lead to the deterioration of LTM for objects and that the hippocampus is required for object memory encoding after long intertrial intervals [73] and persistence of object recognition memory [74]. Thus, we hypothesized that treatment with a cognitive enhancer could result in the maintenance of LTM for objects over time. To evaluate the persistence of LTM (T2), an additional object recognition test session was performed 24 h following the T1 session. Similar to the T1 session, one sample object (object A) and one novel (object C) were placed in the opposite corners of the open field arena (right or left corner) in the T2 session. The session lasted 10 min (Figure 1A–E). Afterwards, the animal was removed from the test environment and returned to the vivarium (n = 10/group).

### 4.5. Molecular Analysis

#### 4.5.1. Total Protein Extraction and Quantification by the Bradford Assay

Twenty-four hours after the T1 (day 8) or T2 (day 9) session ended, the animals (n = 5/group) were euthanized by decapitation. The dHF was rapidly removed, immediately frozen in liquid nitrogen and stored at −80 °C until extraction. Briefly, the samples were homogenized in 1 mL of extraction buffer containing 50 mM Tris HCl, 100 mM NaCl, 50 mM NaF, 1 mM NaVO_4_, 0.5% NP-40, 10 μg/mL leupeptin, 10 μg/mL aprotinin and 0.5 mM PMSF and were incubated with the buffer for 20 min on ice. The sample lysates were centrifuged at 4 °C and 14,000 rpm for 15 min. The supernatants were collected, aliquoted and stored at −80 °C. Sodium orthovanadate (NaVO_4_) was used as a phosphatase inhibitor, and leupeptin, aprotinin and PMSF were used as protease inhibitors. The protein concentrations of the supernatants were quantified with a NanoDrop 200c spectrophotometer (Thermo Scientific, Wilmington, DE, USA) using Bradford reagents (Sigma-Aldrich, St. Louis, MO, USA) according to the manufacturer’s recommendations and bovine serum albumin (BSA) as a standard.

#### 4.5.2. Western Blotting

Homogenates (50μg of protein) were subjected to SDS-PAGE on a 12% polyacrylamide gel (12% acrylamide, 0.1% *w/v* SDS, 0.1% (NH4) 2S2O8, Temed, 0.39 M Tris pH 8.8); 10% of the elution volume of each extract was mixed with 4× buffer (100 mM Tris-HCl pH 6.8, 10% *v/v* 2-mercaptoethanol, 4% *w/v* SDS, 0.02% *w/v* bromophenol blue, and 20% glycerol). Prior to being added to the gel, the samples were denatured by heating at 100 °C for 5 min. The potential difference applied was 200 V, and the current intensity was 150 mA. The running buffer contained 25 mM Tris-base, 250 mM glycine and 0.1% SDS. After electrophoresis, the proteins were transferred to a nitrocellulose membrane in transfer buffer (48 mM Tris, 39 mM glycine, 0.037% SDS and 20% methanol) and subjected to a potential difference of 60 V and a current intensity of 150 mA. The transfer was performed at 4 °C for 3 h. Nonspecific binding was then blocked by incubating the membrane with 5% skimmed milk powder in PBS-T (phosphate-buffered saline, 137 mM NaCl, 10 mM phosphate, 2.7 mM KCl, pH 7.4, and 0.1% Tween) for 1 h at room temperature under constant stirring. After blocking, the membrane was washed (3×) in PBS-T solution for 10 min and cut based on the size of the proteins to be evaluated. For BDNF detection, the membrane was incubated with a BDNF primary antibody (dilution, 1:700) (700 rabbit Abcam^®^, Inc., Cambridge, MA, USA) for 16 h at 4 °C under constant stirring. An anti-α-tubulin antibody) (dilution, 1: 10.000) (Sigma-Aldrich, St Louis, MO, USA) was used as the loading control. After incubation, the membrane was incubated with a conjugated anti-rabbit secondary antibody (Santa Cruz Biotechnology, Santa Cruz, CA, USA) diluted 1: 10.000 in 5% skimmed milk powder in PBS-T for 1 h at room temperature under constant agitation.

Finally, the membrane was washed (3×) times in PBS-T for 10 min. Immunoreactivity was evaluated with the Immobilon Western Chemiluminescent HRP Substrate kit (Millipore, Billerica, MA, USA). The bands were quantified using ImageQuant LAS 4000 (GE, Buckinghamshire, UK). The data were analysed using repeated-measures ANOVA followed by Dunnett’s multiple comparisons.

## 5. Conclusions

Based on the findings from the present study, the persistence of memory is a process associated with mechanisms initiated during consolidation that persist over time. Furthermore, we reveal for the first time that EGb treatment before the acquisition of ORM increases BDNF expression in the hippocampus in a dose-dependent manner, which is associated with the persistence of object recognition memory. This finding was substantiated by the different levels of BDNF expression observed during the T1 and T2 sessions following treatment with 250 or 1000 mg/kg EGb. Conversely, rats treated with 500 mg/kg EGb showed upregulation during the T2 session, which was not sufficient to maintain the storage of recognition memory traces over time.

## Data Availability

The data presented in this study are available on request from the corresponding author.

## Supplementary Materials

The following are available online. Table S1: Recognition Index (RI) values in the retention and persistence tests. Table S2: The levels of rearing and grooming during analysis of long-term memory for objects sessions.

## Sample Availability

Samples of the compounds are not available from the authors.
