# Supplementary material for: BDNF as a Putative Target for Standardized Extract of Ginkgo biloba-Induced Persistence of Object Recognition Memory
Source: Molecules. 2021 Jun 1;26(11):3326. doi: 10.3390/molecules26113326 (PMC8198829; doi:10.3390/molecules26113326)
Supplement: Supplementary file 1 [file molecules-26-03326-s001.zip › molecules-1182931-supplementary.pdf]

Supplementary Material

Table 1. Recognition index (RI) values in the retention and persistence tests.

Groups	1º Minute	2–4 block	5–7 block	8–10 block
**Retention Test**				
Vehicle	0.5938 ± 0.042	0.5955 ± 0.029	0.5249 ± 0.041	0.5472 ± 0.038
4 mg/Kg Diazepam	0.6197 ± 0.046	0.6481 ± 0.026	0.5219 ± 0.036	0.5586 ± 0.033
250 mg/kg EGb	0.6893 ± 0.047	0.6298 ± 0.029	0.6403 ± 0.031	0.6403 ± 0.035
500 mg/kg EGb	0.6691 ± 0.058	0.6163 ± 0.033	0.6074 ± 0.033	0.5752 ± 0.048
1000 mg/kg EGb	0.8178 ± 0.036	0.5296 ± 0.036	0.6383 ± 0.039	0.5004 ± 0.035
**Persistence Test**				
Vehicle	0.5776 ± 0.040	0.5796 ± 0.036	0.5984 ± 0.060	0.6054 ± 0.040
4 mg/Kg Diazepam	0.4823 ± 0.034	0.4730 ± 0.037	0.5469 ± 0.046	0.4897 ± 0.011
250 mg/kg EGb	0.6995 ± 0.053	0.6370 ± 0.040	0.4648 ± 0.093	0.5539 ± 0.034
500 mg/kg EGb	0.6185 ± 0.089	0.6039 ± 0.034	0.5514 ± 0.047	0.6281 ± 0.036
1000 mg/kg EGb	0.7195 ± 0.075	0.6076 ± 0.047	0.5357 ± 0.041	0.5205 ± 0.042

Data (mean ± SEMs) from the control groups (vehicle- and diazepam-treated groups) during the retention test session (n=20/group) and persistence test session (n = 10/group).

Table 2. The levels of rearing and grooming during analysis of long-term memory for objects.

Retention test			
	Diazepam (b)	20.6 ± 1.664	6.6 ± 0.5740
	EGb 250 mg/kg (c)	14.8 ± 1.460	3.8 ± 0.7591
	EGb 500 mg/kg (d)	20.8 ± 1.658	6.8 ± 0.4860
	EGb 1000 mg/kg (e)	14.1 ± 1.562	4.1 ± 0.5262
Persistence test			
	Vehicle (a)	20.6 ± 1.661	6.6 ± 0.8406
	Diazepam (b)	12.5 ± 1.579	4.2 ± 0.5538
	EGb 250 mg/kg (c)	17.6 ± 1.694	4.1 ± 0.2667
	EGb 500 mg/kg (d)	17.7 ± 2.667	3.6 ± 0.5617
	EGb 1000 mg/kg (e)	14.8 ± 3.738	3.4 ± 0.5538

Data (mean ± SEMs) from the control groups (vehicle- and diazepam-treated groups) and the groups treated with EGb (250, 500 and 1000 mg/kg) during the retention test session (n = 20/group) and persistence test session (n = 10/group).
